# Inequality in oral health related to early and later life social conditions: a study of elderly in Norway and Sweden

**DOI:** 10.1186/s12903-015-0005-y

**Published:** 2015-02-10

**Authors:** Ferda Gülcan, Gunnar Ekbäck, Sven Ordell, Stein Atle Lie, Anne Nordrehaug Åstrøm

**Affiliations:** Department of Clinical Dentistry, Faculty of Medicine and Dentistry, University of Bergen, Bergen, Norway; Örebro County Council, Örebro, Sweden; School of Health and Medical Sciences, Örebro University, Örebro, Sweden; Dental Commissioning Unit, Östergötland County Council, Linköping University, Linköping, Sweden

**Keywords:** Life-course perspective, Ageing, OIDP, Tooth loss, Cohort, Social inequality

## Abstract

**Background:**

A life course perspective recognizes influences of socially patterned exposures on oral health across the life span. This study assessed the influence of early and later life social conditions on tooth loss and oral impacts on daily performances (OIDP) of people aged 65 and 70 years. Whether social inequalities in oral health changed after the usual age of retirement was also examined. In accordance with “the latent effect life course model”, it was hypothesized that adverse early-life social conditions increase the risk of subsequent tooth loss and impaired OIDP, independent of later-life social conditions.

**Methods:**

Data were obtained from two cohorts studies conducted in Sweden and Norway. The 2007 and 2012 waves of the surveys were used for the present study. Early-life social conditions were measured in terms of gender, education and country of birth, and later-life social conditions were assessed by working status, marital status and size of social network. Logistic regression and Generalized Estimating Equations (GEE) were used to analyse the data. Inverse probability weighting (IPW) was used to adjust estimates for missing responses and loss to follow-up.

**Results:**

Early-life social conditions contributed to tooth loss and OIDP in each survey year and both countries independent of later-life social conditions. Lower education correlated positively with tooth loss, but did not influence OIDP. Foreign country of birth correlated positively with oral impacts in Sweden only. Later-life social conditions were the strongest predictors of tooth loss and OIDP across survey years and countries. GEE revealed significant interactions between social network and survey year, and between marital status and survey year on tooth loss.

**Conclusion:**

The results confirmed the latent effect life course model in that early and later life social conditions had independent effects on tooth loss and OIDP among the elderly in Norway and Sweden. Between age 65 and 70, inequalities in tooth loss related to marital status declined, and inequalities related to social network increased.

## Background

Globally, the elderly population is growing faster than any other age group [1]. As a consequence of living longer and retaining their natural teeth, older populations have received increasing attention from health policy decision makers [2,3]. A reduction in the rates of tooth loss across time has occurred in many industrialized societies, including the Scandinavian countries [4]. Higher rates of dentate subjects and population ageing imply a continuously increasing demand for and expenditure on oral health care services [2]. Although the Scandinavian countries have generous redistributive policies, absolute and relative inequalities in oral health indicators have been reported to persist in the adult populations across time [3,5-9]. Consistent evidence suggests that people in lower socioeconomic position have worse health and oral health compared with their counterparts in higher socioeconomic position [10]. Little is known about inequalities related to social conditions in oral health of the elderly populations and whether those inequalities remain stable, increase or decrease after the usual age of retirement [10-12].

A life-course perspective to chronic disease epidemiology considers the importance of time in disease development, and offers ways of explaining the social gradient in health by recognizing influences of socially patterned exposures across the entire life span [13,14]. Life course epidemiology has been defined as the study of long-term effects on subsequent health of physical or social exposure during gestation, childhood, adolescence, young adulthood and later adult life [13,14]. According to this perspective, combinations, accumulations and/or interactions of social environments and biological insults experienced throughout the life course impact on current and future health and oral health conditions [13]. The influence of life course exposures on health and oral health has been grouped into various conceptual models that may operate simultaneously; the latent effect model or critical period model, the social mobility or trajectories model and the cumulative life course model being the most frequently investigated [13,15]. According to the latent effect life course model, adverse early-life social conditions increase the risk of chronic disease in later life, independent of subsequent, intervening social circumstances, lifestyle and traditional risk factors. It is assumed that exposures at a specific period during the life span will result in irreversible damage and insult [13,15]. The cumulative life course model considers that risk to health accumulates gradually across the lifespan and focuses on the total amount of exposure, whereas the social mobility model refers to social mobility across the life-course, and to how mobility impacts adult oral health.

Consistent with various life-course models, evidence suggests that deprivation in early-life stages followed by a subsequent affluent status combine to produce elevated cardio-vascular mortality risk [14,16]. Poulton et al. [17] found that early parental socioeconomic position was associated with dental caries at age 26 after adjustment for contemporaneous adult occupational status. Nicolau et al. [18] provided evidence that parental education was related to periodontal health in middle-aged women independent of their contemporaneous educational level. In contrast, results based on the Newcastle Thousand Family study in the UK revealed no association between parental social class and tooth retention at age 50 [19,20]. Åstrøm and Wold [21] investigated how changes in socioeconomic position characteristics throughout adolescent years influenced oral impacts in young adulthood and reported that continuity of an advantaged or disadvantaged socioeconomic position contributed to differing levels of oral health. Thus, participants with stable high socioeconomic position were less likely to report oral impacts at age 30, whereas those with low socioeconomic position were more likely to report oral impacts. Using data from the Health 2000 Survey with a representative sample of Finnish adults, Bernabe et al. [22] investigated the relationship between education and several oral outcomes. They reported results that support the critical period, accumulation and social trajectories models. Whereas the critical period model has received some empirical support [16,23], the life-course perspective on oral health has been criticized for placing too much emphasis on the early life course. This is at odds with the notion that the critical period concept more broadly refers to any stage of an individual’s development during which risk or protective factors may influence health at subsequent life stages. Thus, it has been suggested to include a range of different social condition measures and data from middle adulthood and large prospective studies with various life course models to allow for informed and generalizable statements about the impacts on health and oral health of adults [16]. In a previous Swedish cohort study, Åstrøm et al. [24] found that disadvantaged socio- behavioural characteristics have a long-lasting effect on oral health-related quality of life throughout middle- age life stages. It remains to be ascertained whether inequalities related to social conditions in oral health persist or change with further ageing. Few studies have compared the relative contribution of early and later life course social conditions on dentition status and oral impacts, and investigated whether social inequalities persist, broaden or narrow after the usual age of retirement in non-institutionalized elderly populations.

Focusing cohorts of the elderly in Norway and Sweden from age 65 to age 70, this study assessed the influence of early and later life social conditions on tooth loss and oral impacts on daily performances (OIDP). Whether social inequalities in oral health change during the 5 year follow-up period after the usual age of retirement was also examined. In accordance with “the latent effect life course model”, it was hypothesized that adverse early-life social conditions increase the risk of subsequent tooth loss and impaired OIDP, independent of later-life social conditions. In this study, social condition was defined broadly using measures tapping into work-based and non-work-based components of the corresponding theoretical construct [25].

## Methods

### Study population

The present study is based on data from two cohort studies conducted in Sweden and Norway. The Swedish cohort study started in 1992 focusing a 1942 birth cohort, being resident in the two counties of Sweden. The Norwegian 1942 cohort study was set up as a companion to the ongoing Swedish cohort to enhance co-operation and cross-national comparative research. The Norwegian first wave data collection started in 2007 and was designed to provide directly comparable data with the Swedish study. In both cohorts data are collected by self-administered questionnaires every 5 years, and the study populations are defined by continuously updated versions of the 1942 cohorts in each country. The analysis of the present paper is based on data collection from 2007 and 2012 waves in both countries. The detailed methods of the two cohorts, including number of participants in both survey years as well as number of follow ups have been reported in previous studies [26,27].

In Norway, the final response rate in 2007 was 58.0% (n = 4211), and in 2012 it was 54.5% (n = 3733). Of the cohort members who completed the 2007 survey (n = 4211), a total of 70.0% (n = 2947) responded in 2012. In Sweden, the final response rates were 73.1% (n = 6078) and 72.2% (n = 5697) in 2007 and 2012, respectively. A total of 4862 (80.0% of the cohort members in 2007) participated also in 2012. The ethical considerations in these studies were in accordance with the principles of the Declaration of Helsinki, and written informed consent was obtained from the participants. In Norway, the 2007 and 2012 studies were approved by the Ethics Committee of the Norwegian Social Science Services (NSD) and Regional Committees for Medical and Health Research Ethics (REK), respectively. In Sweden, the first wave study in 1992 was approved by the Ethics Committee in Örebro, and the 2007 and 2012 studies were approved by the Ethics Committee of Uppsala.

### Measures

Data were collected using a structured self-administered questionnaire. To ensure comparability of data, similar questionnaires were administered in the same way across the participating countries. Following the approach of Pearce et al. [19,20], early and later life social conditions assessed in 2007 and 2012 were grouped into a conceptual framework according to the life-course stages at which they would be expected to operate. Gender, country of birth and education, denoted early- life social conditions, were supposed to have been operating from early childhood (gender and country of birth)/early adult life (education), and expected to be time invariant. Marital status, working status and social network denoted later- life social conditions, were expected to occur later in life and to be time variant. *Educational level* was categorized as (1) primary school, (2) secondary school, (3) high school, (4) university/ university college and (5) other. This was dichotomized into (0) higher education (category 4) and (1) lower education (including categories 1, 2, and 3). *Working status* was assessed by asking “how many hours do you work in average per week?” with categories (1) full-time (more than 35 hours/week), (2) part time (between 15 and 34 hours/week), (3) between 1–14 hours and (4) not working. The variable was dichotomized into (0) working (including categories 1, 2 and 3) and (1) not working (category 4). *Marital status* was dichotomized into “married” (category married) and “single” (categories unmarried, divorced and widowed). *Social network* was assessed using the following question “How many people you know, you meet or talk with you during a typical week?” with response alternatives (1) none, (2) 1–2, (3) 3–5, (4) 6–10, (5) 11–15 and (6) more than 15. For analysis, the variable was dichotomized into (0) broad social network (category 6) and (1) narrow social network (including categories 1, 2, 3, 4 and 5).

*Dentition status (tooth loss)* was assessed by asking “How many of your own teeth do you still have (excluding baby teeth)?” with response categories (1) all (28–32 teeth), (2) missing some teeth, (3) missing many teeth, (4) almost no teeth left and (5) edentulous. A dummy variable was constructed (0) all/almost all teeth (including categories 1 and 2) and (1) lost teeth (including categories 3, 4 and 5). In a Norwegian sub-study the measure was validated providing a weighted kappa score of 0.69. Contrary to the Norwegian data, self-reported number of teeth was not validated in the Swedish study group. However, close agreement between the clinically recorded and self-reported number of teeth has been documented previously in the literature [28]. *Oral health-related quality of life* was assessed by the eight-item “Oral Impacts on Daily Performance” (OIDP) frequency inventory [29]. “During the past 6 months, how often have problems with your mouth and teeth caused you any difficulty with: eating and enjoying food; speaking and pronouncing clearly: cleaning teeth; sleeping and relaxing; smiling and showing teeth without embarrassment; maintaining usual emotional state; enjoying contact with people and carrying out major work?” Each item was scored on a 5-point scale, as follows: (1) never affected, (2) less than once a month, (3) once or twice a month, (4) once or twice a week, (5) every/nearly every day. For the purpose of analysis the items were dichotomized into (1) affected (including categories 2–5) and (0) never affected (category 1). A sum score, OIDP frequency SC, was constructed from the 8 dummy performances. OIDP frequency SC (0–8) was dichotomized into (0) no daily performance affected (score 0) and (1) at least one daily performance affected (including score 1 to 8). The OIDP inventory has been tested for psychometric properties previously both in Norway and Sweden [30,31].

### Statistical analysis

All analyses were conducted country wise using SPSS Version 20 (SPSS Inc., Chicago, IL, USA) and STATA version 13.1 (Stata Corporation, College Station, TX, USA). Inverse probability weighting (IPW) was used to adjust estimates for missing responses and loss to follow- up. By IPW, the cohort participants are weighted by the inverse of their probability of being followed- up [32]. Initially, participants and drop-outs were compared on social conditions assessed in 2007 [26]. IPW were estimated by fitting a logistic regression model with variables that contributed to follow- up. The IPW was calculated in the following way: (I) a logistic regression model was fitted for each outcome variable and variables were included in the model to determine whether subjects who remained in the study differed from those lost to follow-up. (II) Based on the estimated model, probabilities were calculated for each participant. (III) Inverse of the probabilities was applied as weights in unadjusted and adjusted logistic regression models. Unadjusted bivariate analyses were performed with the intact cohorts (n = 2947 in Norway and n = 4862 in Sweden) using Cochrane’s Q for repeated measures and cross tabulation with Chi-square tests. For the latent effect life course model, stepwise multiple logistic regression models adjusted using IPW were fitted separately for each survey year and country with odds ratios (OR) and 95% confidence interval (CI). Early-life social condition variables were entered in step 1 and later-life social condition variables in step 2. In each multiple logistic regression model, Nagelkerke’s R^2^ were calculated. Nagelkerke’s R^2^ is a pseudo R square that generalize the coefficient of determination with values between 0 and 1 where 0 denotes that the model do not explain anything about the variation and 1 that the model completely explains variation in the outcome variables. Changes in the association of social conditions with oral health outcomes across time were modelled using Generalized Estimating Equations (GEE) with robust variance estimates to account for the cluster effects of repeated observations.

## Results

In Norway, 74.3% and 67.5% of the non- responders and responders (p < 0.001) reported having lower education. In Sweden, statistically significant differences between respondents and non- respondents occurred regarding foreign country of birth (5.4% versus 9.9%, p < 0.001) and unmarried civil status (67.8% versus 79.2%, p < 0.001) when assessed in 2007 [26].

In Norway, the percentage of tooth loss and oral impacts (OIDP > 0) in 2007 were 21.8% and 23.2%. The corresponding figures in 2012 were 29.0% and 28.4%. In Sweden, the percentage of tooth loss and oral impacts in 2007 were 25.9% and 27.3%, and in 2012 27.3% and 20.4%. Prevalence of being single, unemployed and having narrow social network increased in both countries during the 5 year follow-up (Table 1).

**Table 1:** **Socioeconomic characteristics and oral health indicators by survey year in Norway (n = 2947) and Sweden (n = 4862), based on individuals with complete data**

		**Norway**		**Sweden**	
**Variables**	**Categories**	**2007% (n)**	**2012% (n)**	**2007% (n)**	**2012% (n)**
Gender	Female	48.8 (1415)		51.2 (2489)	
	Male	51.2 (1486)		48.8 (2373)	
Country of birth	Native	98.1 (2822)		94.6 (4520)	
	Foreign	1.9 (56)		5.4 (259)	
Education	Higher	32.5 (770)		24.3 (1027)	
	Lower	67.5 (1601)		75.7 (3192)	
Working status	Working	53.3 (1498)	33.5 (936)	48.7 (2303)	22.3 (1027)
	Not working	46.7 (1314)	66.5 (1858)***	51.3 (2428)	77.7 (3585)***
Marital status	Married	80.4 (2314)	77.3 (2265)	79.2 (3781)	76.0 (3524)
	Single	19.6 (565)	22.7 (667)***	20.8 (995)	24.0 (1114)***
Social network	Broad	38.8 (1115)	21.9 (635)	39.8 (1880)	25.6 (1185)
	Narrow	61.2 (1758)	78.1 (2267)***	60.2 (2849)	74.4 (3441)***
Tooth loss	All or almost all teeth	78.2 (2224)	76.8 (2164)	74.1 (3515)	72.7 (3404)
	Lost teeth	21.8 (619)	23.2 (655)***	25.9 (1230)	27.3 (1276)***
OHRQoL	OIDP = 0	71.0 (1975)	71.6 (2002)	72.7 (3375)	79.6 (3654)
	OIDP > 0	29.0 (806)	28.4 (796)	27.3 (1269)	20.4 (935)***

Cochrane’s Q-test: *p < 0.05, **p < 0.01, ***p < 0.001.

The total number in the different categories do not add up to 2947 due to missing values.

Information regarding some parts of the table is present elsewhere [26].

Table 2 depicts the percentage of participants having major tooth loss and OIDP > 0 by early and later life social conditions separately for each survey year and country. Educational level, working status, marital status and social network were statistically significantly related to tooth loss. In Norway, gender was associated with OIDP in 2007, whereas both gender, country of birth and marital status were associated with OIDP in 2012. In Sweden, oral impacts (OIDP > 0) was reported by 26.5% of participants of native Swedish origin and by 39.5% of participants with foreign country origin. Corresponding figures in 2012 were 19.8% versus 29.5%. Marital status and social network were statistically significantly associated with oral impacts in 2007 and 2012.

**Table 2:** **Percentage (n) tooth loss and OIDP (OIDP > 0) by early and later life social conditions in 2007 and 2012, in Norway (n = 2947) and Sweden (n = 4862), based on individuals with complete data**

	**Norway**		**Sweden**	
	**Tooth loss% (n)**	**OIDP > 0% (n)**	**Tooth loss% (n)**	**OIDP > 0% (n)**
**2007**				
**Early-life social conditions**				
Female	21.2 (288)	26.9 (356)	26.1 (632)	28.1 (664)
Male	22.4 (326)	30.9 (444)*	25.7 (598)	26.6 (605)
Native	21.8 (598)	28.7 (771)	24.8 (1101)	26.5 (1154)
Foreign	24.1 (13)	41.2 (21)	46.1 (117)***	39.5 (96)***
Higher education	11.7 (88)	27.4 (204)	17.9 (182)	29.4 (289)
Lower education	27.3 (423)***	29.8 (451)	28.8 (901)***	26.2 (808)
**Later-life social conditions**				
Working	19.5 (284)	29.3 (419)	23.2 (525)	25.5 (562)
Not working	24.4 (311)**	28.8 (361)	28.4 (679)***	28.9 (679)**
Married	20.5 (460)	28.5 (631)	23.8 (888)	25.3 (927)
Single	27.5 (150)***	30.6 (160)	33.8 (329)***	34.7 (325)***
Broad social network	17.7 (191)	25.5 (270)	22.3 (411)	24.7 (446)
Narrow social network	24.6 (421)***	31.4 (526)**	28.1 (787)***	29.0 (796)**
**2012**				
**Early-life social conditions**				
Female	21.3 (286)	25.2 (335)	27.3 (649)	20.2 (467)
Male	25.0 (358)*	31.5 (450)***	27.3 (627)	20.6 (468)
Native	23.1 (623)	28.3 (759)	26.2 (1145)	19.8 (848)
Foreign	31.5 (17)	40.7 (22)*	46.6 (115)***	29.5 (70)***
Higher education	12.6 (94)	26.8 (199)	19.5 (195)	20.1 (200)
Lower education	28.6 (434)***	29.9 (452)	29.6 (909)***	19.9 (600)
**Later-life social conditions**				
Working	21.4 (192)	27.5 (244)	25.0 (251)	21.1 (208)
Not working	23.6 (421)	29.0 (513)	27.5 (964)	19.8 (681)
Married	21.7 (472)	27.2 (589)	25.4 (875)	18.7 (635)
Single	28.3 (180)***	32.4 (202)**	32.1 (348)***	24.7 (260)***
Broad social network	16.8 (103)	25.3 (154)	21.5 (251)	19.4 (222)
Narrow social network	25.1 (543)***	29.3 (631)	29.1 (977)***	20.4 (670)

Chi-square: *p < 0.05, **p < 0.01, ***p < 0.001.

Modelling tooth loss and OIDP using multiple logistic regression adjusted with IPW, early-life social condition indicators in terms of gender, country of birth and educational level were entered in a first step, followed in a second step by later-life social condition indicators; working status, marital status and social network. In Norway, the final logistic model for tooth loss provided a Nagelkerke’s R^2^ of 0.05 in 2007 and 2012. The corresponding figures for OIDP were 0.01 in 2007 and 2012. In 2007, major tooth loss were more likely to occur among males (OR = 1.3), lower educated (OR = 2.7), unemployed (OR = 1.5), and single people (OR = 1.5) (Table 3). In 2012, males, lower educated, single people and those with narrow social network were more likely to report major tooth loss. The corresponding ORs were 1.6, 2.5, 1.6 and 1.7. GEE analyses revealed a statistically significant two-way interaction between social network and survey year (time) on tooth loss, OR = 1.4 (95% CI 1.1-1.9). The ORs for having tooth loss if having a narrow network increased statistically significantly from OR = 1.2 in 2007 to OR = 1.7 in 2012 (p < 0.05) (Table 3). In Sweden the final regression models for tooth loss provided Nagelkerke’s R^2^ of 0.03 and 0.02 in 2007 and 2012, respectively. Nagelkerke’s R^2^ for OIDP were 0.01 for both survey years. Country of birth and education were the most important early-life social condition predictors of tooth loss across the survey years. Working status, marital status and social network were the most important later-life social condition predictors of tooth loss in 2007 and 2012, respectively. A two-way interaction on tooth loss between survey year and marital status occurred, OR = 0.8 (95% CI 0.7-0.9). The ORs declined from 1.6 in 2007 to 1.3 in 2012 (p < 0.05) (Table 3).

**Table 3:** **Early and later life social conditions and two way interactions between social conditions and time regressed on tooth loss in Norway (n = 4211) and Sweden (n = 6078)**

	**2007 OR (95% CI)** ^**a**^	**2012 OR (95% CI)** ^**b**^	**Interaction social condition X time OR (95% CI)** ^**c**^
**Norway**			
**Early-life social conditions**			
Male vs female	1.3 (1.1-1.6)	1.6 (1.3-2.0)	1.2 (0.9-1.5)
Foreign vs native	1.2 (0.7-2.2)	1.6 (0.8-3.3)	1.3 (0.6-2.5)
Lower vs higher education	2.7 (2.2-3.3)	2.5 (1.9-3.3)	0.9 (0.7-1.2)
**Later-life social conditions**			
Not working vs working	1.5 (1.2-1.8)	1.2 (0.9-1.5)	0.8 (0.6-1.1)
Single vs married	1.5 (1.2-1.8)	1.6 (1.2-2.0)	1.1 (0.8-1.4)
Narrow vs broad social network	1.2 (1.0-1.5)	1.7 (1.3-2.3)	1.4 (1.1-1.9)
**Sweden**			
**Early-life social conditions**			
Male vs female	1.1 (0.9-1.2)	1.0 (0.9-1.2)	0.9 (0.8-1.1)
Foreign vs native	2.1 (1.7-2.7)	2.5 (1.8-3.4)	1.2 (0.9-1.6)
Lower vs higher education	1.9 (1.6-2.2)	1.9 (1.6-2.3)	1.0 (0.8-1.2)
**Later-life social conditions**			
Not working vs working	1.2 (1.1-1.4)	1.1 (0.9-1.3)	0.9 (0.7-1.1)
Single vs married	1.6 (1.4-1.8)	1.3 (1.1-1.5)	0.8 (0.7-0.9)
Narrow vs broad social network	1.3 (1.1-1.5)	1.4 (1.2-1.7)	1.1 (0.9-1.4)

Adjusted OR and (95% CI).

a) Logistic regression showing main effects of social conditions in 2007 on tooth loss in 2007. Inverse probability weighting (IPW) adjusted estimates.

b) Logistic regression showing main effects of social conditions in 2012on tooth loss in 2012. Inverse probability weighting (IPW) adjusted estimates.

c) Results from GEE showing two way interactions between time and social conditions on tooth loss indicating change in associations from 2007 to 2012.

With respect to OIDP, gender and social network were the only statistically significant early and later life social condition predictors in Norway in 2007 (Table 4). Compared to females, males were more likely to report OIDP. People having narrow social network were more likely than their counterparts with large social network to report oral impacts. In 2012, gender and educational level were significant early-life social condition predictors of OIDP, whereas marital status was the only later-life social condition predictor. In Sweden, participants of foreign country of birth, those with single marital status and narrow social network were more likely to report OIDP in 2007. Country of origin and marital status were significant early and later life social conditions predictors of OIDP in 2012 (Table 4). GEE revealed no statistically significant two- way interactions between early and later life social condition indicators and time on OIDP in either country (Table 4).

**Table 4:** **Early and later life social conditions and two way interactions between social conditions and time regressed on OIDP in Norway (n = 4211) and Sweden (n = 6078)**

	**2007 OR (95% CI)** ^**a**^	**2012 OR (95% CI)** ^**b**^	**Interaction social condition X time OR (95% CI)** ^**c**^
**Norway**			
**Early-life social conditions**			
Male vs female	1.3 (1.1-1.5)	1.5 (1.3-1.9)	1.2 (0.9-1.4)
Foreign vs native	1.4 (0.9-2.4)	1.2 (0.6-2.4)	0.8 (0.4-1.8)
Lower vs higher education	1.1 (0.9-1.3)	1.2 (1.0-1.5)	1.1 (0.9-1.4)
**Later-life social conditions**			
Not working vs working	1.0 (0.9-1.2)	1.1 (0.9-1.3)	1.1 (0.8-1.4)
Single vs married	1.1 (0.9-1.4)	1.4 (1.2-1.8)	1.3 (0.9-1.6)
Narrow vs broad social network	1.3 (1.1-1.5)	1.1 (0.9-1.4)	0.9 (0.7-1.2)
**Sweden**			
**Early-life social conditions**			
Male vs female	1.0 (0.9-1.2)	1.0 (0.9-1.2)	1.0 (0.8-1.2)
Foreign vs native	1.5 (1.2-2.0)	1.6 (1.1-2.3)	1.0 (0.7-1.5)
Lower vs higher education	0.8 (0.7-1.0)	0.9 (0.8-1.2)	1.2 (0.9-1.4)
**Later-life social conditions**			
Not working vs working	1.1 (0.9-1.2)	1.0 (0.8-1.2)	0.9 (0.7-1.1)
Single vs married	1.5 (1.3-1.8)	1.4 (1.2-1.7)	0.9 (0.8-1.2)
Narrow vs broad social network	1.3 (1.1-1.5)	1.1 (0.9-1.3)	0.8 (0.7-1.1)

Adjusted OR and (95% CI).

a) Logistic regression showing main effects of social conditions in 2007 on OIDP in 2007. Inverse probability weighting (IPW) adjusted estimates.

b) Logistic regression showing main effects of social conditions in 2012on ODIP in 2012. Inverse probability weighting (IPW) adjusted estimates.

c) Results from GEE showing two way interactions between time and social conditions on OIDP indicating change in associations from 2007 to 2012.

## Discussion

Few population-based prospective cohort studies have investigated social inequalities in self- reported oral health of older people across societies belonging to the same welfare regime [3,10,11,33]. This study examined inequalities in tooth loss and oral impacts on daily performances (OIDP) related to early and later life social conditions focusing on non- institutionalized Norwegian and Swedish elderly. Although 65 years of age is recognized as the norm for retirement in Norway and Sweden, many continue to work until older ages [34,35]. Evidence suggests that higher educated and married people tend to retire after age 65 more frequently than their single counterparts with lower-level education [34,35]. In both countries, being at work after age 65 may reflect social differences in terms of educational level, perceived health, occupational status and working environment.

Across countries, major tooth loss and OIDP at ages 65 and 70 were more prevalent among those with lower social-condition categories, independent of how social condition was measured. The latent effect life-course model was supported in that both early and later life social conditions had independent effects on tooth loss and OIDP. Moreover, with few exceptions, social inequalities in major tooth loss and oral impacts remained stable across the survey years. These results corroborate previous population based studies reporting consistent education and income gradients in clinical and subjective oral health indicators similarly to respective social gradients in general health [3,4,10,11,21]. National health surveys have reported that considerable social inequalities in health are present in European countries [36,37]. In spite of their emphasize put on egalitarian principles, Norway and Sweden being no exception in this respect [36,37].

Consistent with previous studies, the present findings indicate that disadvantage in early life would have an enduring detrimental effect on future health, irrespective of intervening later life experiences [13]. A cohort study from United Kingdom demonstrated persistent influence of early-life social conditions on tooth loss at age 50 [19]. In a Danish study [38], early-life social conditions in terms of higher education predicted higher number of filled teeth at age 85, suggesting that well educated people seek dental care more frequently than their lower educated counterparts. Bernabe et al. [22] showed that both parental and own education contributed independently to adult oral health among Finnish adults. The results of the present study corroborate evidence suggesting that early-life social conditions influence mortality and chronic diseases at older ages [16]. In addition to the cross-sectional analyses in 2007 and 2012, GEE was utilized to examine whether changes occurred in the social inequalities of major tooth loss and oral impacts from 2007 (age 65) to 2012 (age 70). The results revealed a significant increase in social network related inequality of tooth loss in Norway and a significant decrease in marital status related inequality of tooth loss in Sweden. Previous studies have shown that social differentials in mortality based on employment and occupational status tend to decrease with increasing age after retirement, whereas inequalities based on social structural measures such as social support and marital status seem to either persist or decrease marginally [39]. This study supports previous conflicting evidence from longitudinal studies, suggesting both persisting, increasing and declining social inequalities in oral health with increased age in Norway and Sweden.

Few studies have provided evidence of educational gradients in broad subjective oral health measures. Contrary to the present results of no significant relationship between education and OIDP, Tsakos et al. [40] found a clear educational gradient in oral impacts as measured by the Geriatric Oral Health Assessment Index; the lower the educational level the worse the oral health perceptions. An inverse graded association between education and oral impacts on daily performances was also reported from the English Longitudinal Survey of Aging, but only among dentate individuals [10]. Cross-national studies have revealed morbidity to be most prevalent among lower educated younger and higher educated older individuals [22,41]. Nevertheless, the lack of an educational gradient in oral impacts as observed in this study is inconsistent with previous studies focusing broad subjective measures of oral health [10,21]. This might be attributed to differences in study populations with various cultural background and the type of educational measures utilized. It has been recognized that education could be a poor measure of material wealth due to different social meanings attached to this concept across time and cultures [42].

Whereas Norway has several social security and welfare benefits by which particular population subgroups have their dental care expenses refunded, Sweden implements benefit schemes of a more universal nature. In spite of between country difference when it comes to inclusiveness of social assistance, the country specific analyses of this study suggest that social inequalities in oral health of the elderly were as profound in Sweden as in Norway [43]. The present results are in keeping with previous ones, suggesting that cross-national variation in health inequalities are smaller within than between various welfare regimens [36,37]. In this study problems making between country comparisons were avoided by similarities in sampling frames, survey questions and the distribution of responders across social condition categories. Nevertheless, the results should still be interpreted by caution since the social meaning of the various social condition groups (e.g. educational level) might vary slightly across study sites. Moreover, the choice of social indicator may have an influence on the disparity estimates presented. Previous studies have shown that social factors related to wealth and prestige may be more sensitive indicators than income and occupational status among older people [44]. Consistent with this evidence, structural measures, such as education, marital status and social network were among the strongest predictors of tooth loss and OIDP in this study.

Some weaknesses of the present study should be considered. In cohort studies, selection biases may arise from unwillingness to participate, missing information and losses to follow-up. Thus, this study had limitations first and foremost in terms of the rates of non-response and losses to follow-up that occurred across time in both countries. Compared to individuals retained across the survey years, those lost to follow-up tended to be disadvantaged in terms of early and later life social condition indicators and also regarding the oral health outcomes investigated. Previous studies have shown that being single is correlated with migration out of the study area which is consistent with the present finding that married were more likely to retain in the survey than non-married [45]. Consistent with the present results, higher educational level is a predictor of missing in cohort studies [33,45]. Although it has been acknowledged that failure to correct for nonresponse in cohort studies produces biases in self- reported health, the IPW attached to subjects included in the analysis may have restored representation of those lost to follow-up and thus reinforced the internal and external validity of the study [32]. Exclusion of institutionalized elderly is another problem that may have led to selection bias since institutionalized people tend to have lower socioeconomic position and are less healthy than their non- institutionalized counterparts [46]. However, this bias has been marginal as institutionalized people in Norway and Sweden are usually above 80 years. The rate of the population between 65 and 74 that are institutionalized is below 5% [47]. Although it is not possible to conclude whether social inequalities in oral health can be attributed to health selection or social causation, previous studies have shown that health selection explains only a minor portion of the observed social gradient in health [48].

## Conclusion

The results confirmed the latent effect life course model in that early and later life social conditions had independent effects on tooth loss and OIDP among the elderly in Norway and Sweden. Social inequalities in oral health remained stable after the usual age of retirement at age 65. Inequalities in tooth loss related to social network and marital status increased and declined from age 65 to 70. The results are important for public oral health decision makers who plan strategies for optimal oral health and quality of life in the older population.

## Abbreviations

OIDP: Oral impacts on daily performances
GEE: Generalized estimating equations
IPW: Inverse probability weighting
